# Antiestrogens Inhibit Xenoestrogen-Induced Brain Aromatase Activity but Do Not Prevent Xenoestrogen-Induced Feminization in Japanese Medaka (*Oryzias latipes*)

**DOI:** 10.1289/ehp.8211

**Published:** 2005-10-27

**Authors:** Adam J. Kuhl, Marius Brouwer

**Affiliations:** 1 University of Southern Mississippi, Ocean Springs, Mississippi, USA; 2 CIIT Centers for Health Research, Research Triangle Park, North Carolina, USA

**Keywords:** brain aromatase, fadrozole, medaka, *o,p*′-DDT, sex differentiation, sex reversal, tributyltin

## Abstract

In fish, exposure to estrogen or estrogen-mimicking chemicals (xenoestrogens) during a critical period of development can irreversibly invert sex differentiation. In medaka, a male-to-female reversal upon exposure to a xenoestrogen is accompanied by an increase in brain aromatase expression and activity. However, whether this increase is the direct cause of sex reversal is unknown. In this study we further examined the role brain aromatase plays in genesis of developmental abnormalities in response to endocrine-disrupting chemicals (EDCs). Further, the effects of a mixture of apparent antagonistic environmentally relevant EDCs on development were examined to determine if their combined actions could lessen each other’s impacts. To this end, hatchling medaka were subjected in a 2-week flow-through immersion exposure to an estrogen mimic [dichlorodiphenyltrichloroethane (*o,p*′-DDT)] and to pharmaceutical [fadrozole (FAD)] and environmental aromatase inhibitors [tributyltin (TBT)] alone and in combination. Brain aromatase expression and enzyme activity were measured on exposure days 5, 9, and 14 by real-time reverse-transcriptase polymerase chain reaction and tritiated water release assay, respectively. We recorded sex reversals at sexual maturity by examining the phenotypic and genotypic sex of d-rR–strain medaka. Results indicate that FAD and TBT inhibit aromatase activity in *o,p*′-DDT–treated fish but do not prevent feminization, indicating that increased brain aromatase activity is not critical to EDC-induced male-to-female sex inversion. The observation that estradiol biosynthesis inhibitors do not block the effect of the xenoestrogen suggests that in the environment, exposure to seemingly antagonistic EDCs does not necessarily lessen the harmful impacts of these compounds.

Beginning early in sex determination and gonadogenesis in fish, communication between nonadjacent tissues is necessary. This communication is accomplished through the endocrine system, which controls sex differentiation through complex interactions between the central nervous system and gonads using pituitary-derived gonadotropins and sex steroids produced in the gonad and brain (Nagahama 1994). Production of these sex steroids is very strongly linked with the early steps of gonadal differentiation, and they can influence long-term sex determination choices (Devlin and Nagahama 2002). Further, it has been demonstrated that exposure to estrogen or estrogen-mimicking chemicals during a critical period of development can result in genotypic XY males developing into fully functional phenotypic females, whereas exposure of genotypic XX females to androgenic chemicals can result in development of phenotypic males (Cheek et al. 2001a, 2001b; Edmunds et al. 2000; Gronen et al. 1999; Hunter and Donaldson 1983; Yamamoto 1969).

Because of the critical role of estrogen in the very early stages of sex determination and sex differentiation, the estrogen-synthesizing enzyme is of likely importance. As in all vertebrates, in fish this function is performed by cytochrome P450 aromatase, which converts androgens to estrogens and is expressed in a number of tissues, including the brain, liver, and gonads. Recently, a second isoform of aromatase (*cyp19b*; Genbank accession no. AY319970; National Center for Biotechnology Information, National Library of Medicine, Bethesda, MD) has been found in the brain of several teleost species (Callard and Tchoudakova 1997; Chiang et al. 2001; Kuhl et al. 2005). This isoform has much higher activity and mRNA levels in the brain than the ovary has of ovarian aromatase (Kishida and Callard 2001).

It has been hypothesized that altered expression of aromatase is important in environmentally influenced sex differentiation. Increases in brain aromatase expression occur in 0- to 14-day-posthatch (dph) medaka concomitantly with xenoestrogen-induced feminization (Kuhl et al. 2005), whereas ovarian aromatase mRNA transcripts at this life stage are not detectable. Conversely, inhibition of aromatase activity can result in masculinization of genotypic females (Piferrer et al. 1994). Whether altered aromatase activity is directly responsible for sex reversal is not known. In the present study we aimed to further examine the critical role aromatase plays in the genesis of developmental abnormalities in response to endocrine disruptors.

Most research to date on the effects of endocrine-disrupting chemicals (EDCs) has focused on abnormalities induced by exposure to a single compound. However, in the environment, humans and wildlife are exposed to diverse mixtures of androgenic, estrogenic, antiandrogenic, and antiestrogenic compounds (Bush et al. 1990). Only recently has focus begun to shift to examining the impact of mixtures of EDCs on human and wildlife populations. Most of this research, however, has focused on the synergism between several weak estrogenic compounds (Bergeron et al. 1999; Payne et al. 2000). These studies demonstrate that there is an additive effect of several weakly estrogenic EDCs. To date, little work has been done on the impacts of mixtures of antagonistic EDCs. It is unknown if an androgenic or anti-estrogenic chemical can block the activity of an estrogenic one. Here we address this question by examining if xenoestrogen [dichloro-diphenyltrichloroethane (*o,p*′-DDT)]-induced feminization of developing medaka can be prevented by coexposure to a pharmaceutical [fadrozole (FAD)] and an environmental aromatase inhibitor (AI), tributyltin (TBT). We hypothesize that *o,p*′-DDT activates the estrogen receptor to induce transcription of brain aromatase. Increased brain aromatase activity results in increased estradiol levels feminizing the development of the fish. We further hypothesize that inhibiting aromatase activity will prevent a xenoestrogen-induced surge of estradiol production and prevent feminization.

*o,p*′-DDT is a known xenoestrogen that can negatively affect reproduction and development in fish, through immersion exposure (Cheek et al. 2001a, 2001b) and direct injection into oocytes (Edmunds et al. 2000). FAD has been used extensively in breast cancer research as a pharmaceutical inhibitor of aromatase and has been shown to be a reversible competitive inhibitor of aromatase in birds (Elbrecht and Smith 1992), mammals (Steele et al. 1987), and fish (Afonso et al. 1999, 2001). In Nile tilapia (*Oreochromis niloticus*), the suppression of aromatase with a FAD-treated diet resulted in a 100% male population Afonso 2001; Kwon et al. 2000). TBT has been used as an antifouling biocide in paint for boats, for wood treatment and preservation, and as a fungicide/bactericide in textile and industrial water systems (Gehring et al. 1991). At very low concentrations, TBT can disrupt the endocrine system, as evidenced by the induction of male sexual characteristics in female gastropods (Oberdorster and McClellan-Green 2002; Smith 1981). TBT has also been shown to be an EDC in fish, as well. A 30-day exposure to 100 ng/L TBT in zebrafish (*Danio rerio*) resulted in an almost completely male population (McAllister and Kime 2003). It is hypothesized that TBT acts as a competitive inhibitor of aromatase, which causes accumulation of testosterone and masculinization of the organism (McAllister and Kime 2003; Oberdorster and McClellan-Green 2002).

Our results from the present study demonstrate that an increase in aromatase activity is not needed for EDC-induced feminization and that antiestrogenic AIs are unable to prevent xenoestrogen-induced feminization.

## Materials and Methods

### Experimental animals.

Medaka (d-rR strain) used in this study were hatched from brood-stock cultured and maintained at the Gulf Coast Research Laboratory, University of Southern Mississippi (Ocean Springs, MS). The d-rR strain contains a Y-chromosome–linked gene coding for a red body color phenotype, allowing for the simple determination of sex genotype: males have red phenotype, and females have white. Medaka also have secondary sex characteristics that are reflective of sexual phenotype. Females have shorter anal fins, and males have a notched dorsal fin. With the d-rR strain and secondary sex characteristics (fin morphology), sex reversals can be determined by simple observation of body color and fin development. Animal care and experimentation were conducted in accordance with University of Southern Mississippi guidelines for animal care and use, and animals were treated humanely with regard for alleviation of suffering. Broodstock cultures were maintained in 300-L fiberglass runs at a 27 ± 1°C. A 16-hr light/8-hr dark photoperiod was provided by timer-controlled overhead fluorescent lights. Broodstock cultures were fed a minimum of twice daily, one feeding of commercial flake (Ziegler Brothers, Santa Anna, CA) and one feeding of brine shrimp nauplii, *ad libitum*.

### EDC exposure.

Medaka were exposed in two consecutive experiments. In the first experiment medaka were exposed to nominal concentrations of 0, 10, 50, and 100 μg/L FAD and 0.7 μg/L TBT. This was designed as a range-finding study to determine potential masculinizing concentrations of a pharmaceutical AI and determine the responsiveness of the system to an environmental AI. The second exposure consisted of nominal concentrations of 300 μg/L FAD, 1.5 μg/L TBT, 7.5 μg/L DDT, 50 μg/L FAD with 7.5 μg/L DDT, 300 μg/L FAD with 7.5 μg/L DDT, and 1.5 μg/L TBT with 7.5 μg/L DDT. DDT concentrations were selected based on levels previously shown to induce male-to-female sex inversion (Kuhl et al. 2005).

Eggs were collected from 15-cm cylindrical filter sponges used as spawning substrates. Embryos were transferred to glass hatching jars containing about 4 L of hatching solution (1.00 g/L NaCl, 0.030 g/L KCl, 0.040 g/L CaCl_2_, 0.162 g/L MgSO_4_ in distilled water) with the salinity of the hatching solution brought to 5 g/L with NaCl to control fungus. Hatching jars were maintained under continuous fluorescent light in a water bath at 24 ± 1°C and vigorously aerated to suspend embryos.

At hatch, 75 d-rR fry were housed in three retention chambers (100-mm Petri dish bottoms with attached 475-μm nylon collar) with 25 fish in each chamber. For the first exposure, fry were exposed to four duplicated exposure treatments (control, three FAD concentrations, and one TBT concentration) for a total of 8 test aquaria. For the second exposure, fry were exposed to seven duplicated exposure treatments (control, carrier control, FAD, two FAD + DDT combinations, TBT, and TBT + DDT) for a total of 14 test aquaria. Test aquaria were 35 L with a water depth of 19 cm maintained by drain siphon. Test aquaria were housed within a central water bath kept at 27 ± 1°C and provided with a 16-hr light/8-hr dark photoperiod supplied by fluorescent bulbs.

Exposure was conducted in a setup similar to that described by Walker et al. (1985) and Manning et al. (1999). Briefly, a water partitioner delivered 2 L of test solution each cycle to splitter/mixing boxes that dispensed 1 L to each duplicate aquarium. The exposure system cycled between three and five cycles per hour during the exposure period. Test concentrations were prepared each cycle by injection of appropriate stock to the splitter boxes of each treatment using Hamilton PSD2 liquid injectors (Hamilton Company, Reno, NV). Stocks were created by dissolving the compound in the appropriate solvent. DDT and TBT were dissolved in triethylene glycol, and FAD was dissolved in well water.

Water quality (pH, temperature, and dissolved oxygen) was measured twice each week, and water samples were removed four times (day 0, 5, 8, and 14) for analytical analysis of the test chemicals. Survival was monitored and recorded daily, and all dead fry were removed. Six fish per aquaria (12/treatment) were sampled, weighed, and archived for molecular analysis on days 5, 9, and 14. Six sampled fish per treatment were preserved in 200 μL RNAlater (Ambion, Austin, TX) for mRNA analysis, and six were preserved in 200 μL phosphate buffer (100 mM KCl, 10 mM KH_2_PO_4_, 1 mM EDTA, 10 mM dithiothreitol, pH 7.4) for enzyme activity analysis. Upon exposure completion, fry were transferred to 18.5-L grow-out aquaria until sexual maturity so secondary sex characteristics could be observed. After sex determination, fish were terminally anesthetized with MS-222 and discarded.

### Real-time quantitative RT-PCR.

Ovarian (*cyp19a*) and brain aromatase (*cyp19b*) expression was measured using real-time quantitative reverse-transcriptase polymerase chain reaction (RT-PCR). Due to lack of detection of ovarian aromatase with real-time PCR, further examination of *cyp19a* was performed using multiple primer pair with visualization of expression using both real-time methods and 2% agarose gel/ethidium bromide. Total RNA was extracted from whole fry using a Trizol procedure and purified with a phenol: chloroform extraction followed by an ethanol precipitation. Total RNA concentration was measured using a Beckman (Fullerton, CA) DU640 spectrophotometer and treated with DNase H (Invitrogen, Carlsbad, CA) to remove genomic DNA contamination. cDNA was synthesized from 1 μg total RNA using Superscript II reverse transcriptase from Invitrogen and random decamers. Real-time PCR was accomplished using Taqman chemistry (Heid et al. 1996).

Forward and reverse primers for *cyp19a* and *cyp19b* amplification and dual dye-labeled FAM (6-carboxyfluorescein; excitation, 490 nm; emission, 520 nm)–Black Hole Quencher (BHQ) were designed from the ovarian and brain aromatase sequence (Genebank accession nos. D82968 and AY319970) using Beacon Designer 3.01 (PREMIER Biosoftware, Palo Alto, CA) (oligo 1–7) (Table 1). 18S primers designed from published medaka 18S sequence and Cy5 (excitation, 596 nm; emission, 615 nm)–Iowa Black RQ dual-labeled Taqman probes (Integrated DNA Technologies, Coralville, IA) were used as internal normalization standard (oligo 8, 9, 10) (Table 1). Integrated DNA Technologies supplied probes, and we used a Bio-Rad (Hercules, CA) IQ-Cycler real-time PCR system to amplify and measure fluorescence of aromatase and 18S. For the reactions in the first exposure, conditions consisted of 100 nM probe, 900 nM primer for aromatase, and 100 nM probe and 50 nM primers for 18S for all sampling days. Primer concentrations were tested to ensure equal amplification efficiency between aromatase and 18S.

Due to differences in the ratio of 18S and aromatase concentrations in fish collected at each sample period in the second exposure, we used different concentrations of probe and primers to obtain equal amplification efficiencies. Day 5 conditions consisted of 100 nM probe, 1,200 nM primer for aromatase, and 100 nM probe and 35 nM primers for 18S. Day 9 conditions were 100 nM probe, 1,200 nM primer for aromatase, and 100 nM probe and 40 nM primers for 18S. On day 14, no primer concentrations could be determined that would express both aromatase and 18S for all samples in multiplex with equal amplification efficiency. Therefore, day 14 samples were measured in separate single-plex reactions using Bio-Rad IQ Real-Time SYBRMix with SYBRGreen. Multiplex reactions used Bio-Rad IQ Real-Time Supermix according to manufacturer’s instructions. Cycle parameters were 50°C for 120 sec, 95°C for 120 sec, 50 cycles of 95°C for 15 sec, and 61°C for 30 sec. Relative expression was calculated with the comparative *C_t_* (Δ Δ*C_t_*) method, which involves comparing the thresh-old cycle (*C_t_*) values of the treated samples with the nontreated controls (calibrator). The *C_t_* values of both the calibrator and the treated samples are then normalized to the endogenous housekeeping gene 18S. Gene expression for each sampling time is expressed as fold increase over same-day control.

### Aromatase activity.

Aromatase activity was measured by a tritiated water release assay based on the work of Thompson and Siiteri (1974) as adapted to medaka by Melo and Ramsdell (2001) and Contractor et al. (2004). Whole medaka fry sampled during exposure were homogenized in phosphate buffer (1 M KCl, 0.01 M K_2_HPO_4_, and 0.001 M EDTA, pH 7.4). Protein concentration of homogenate was determined using a bicinchoninic acid protein assay kit (Pierce, Rockford, IL). Homogenate containing about 20 mg of protein was incubated with 5 nM androst-4-ene-3,17-dione [1β-^3^H(N)] (Perkin Elmer, Boston, MA) in a 200 μL solution of 1 mM NADPH, 10 mM glucose-6-phosphate, 1 U/mL glucose-6-dehydrogenase, 10 mM potassium phosphate (dibasic), 1,000 mM potassium chloride, 1 mM EDTA, and 1 mM dithiothreitol at 37°C for 3 hr. After incubation, reactions were terminated by immersion in ice-cold water and adding 100 μL 30% trichloroacetic acid, and centrifuged at 1,700*g* for 10 min to remove precipitated protein. Unconverted substrate was removed by vortexing vigorously for 60 sec with 1 mL chloroform followed by centrifugation at 1,700*g* for 25 min at 4°C. Addition of a 5% charcoal/0.5% dextran slurry followed by a 40 sec vortex and 30 min centrifugation (10,000*g*) was used to remove any residual androst-4-ene-3,17-dione. Radioactivity of tritiated water was measured in a Beckman LS6500 liquid scintillation counter and background subtracted using samples without homogenate. Sensitivity of detection was set at two standard deviations above the mean blank activity to be considered detectable.

### Analytical chemistry.

Magnolia Scientific Services Inc. (Hattiesburg, MS) measured *o,p*′-DDT and TBT. *o,p*′-DDT was measured according to U.S. Environmental Protection Agency (EPA) method 608 (U.S. EPA 1988), and TBT was measured according to U.S. EPA method 282.3 (U.S. EPA 1989).

We determined FAD concentration using reverse-phase high-pressure liquid chromatography (HPLC) with photo diode array detection. Water samples (~ 5 mL) were collected twice weekly in glass vials and injected directly as 1.0 mL aliquots onto a 4.6 mm × 25 cm Beckman Ultrasphere C18 reverse-phase column connected to a Beckman Gold HPLC system. Samples were chromatographed using a gradient program with a mobile phase starting at 60% methanol/40% 50 mM phosphate buffer (pH 7.0) for 4 min and increased to 80% methanol/20% buffer over 2 min, where it was held for 9 min. Mobile phase was then returned to 60% methanol/20% phosphate buffer to prepare the column for the next sample. FAD in column eluate was detected using a Beckman System Gold 168 photodiode array detector set at 229 nm. A five-point standard curve was developed with FAD dissolved in well water for sample quantification. Samples were measured in duplicate in conjunction with standards. Limit of detection was approximately 5 μg/L.

### Statistical analysis.

We compared treatments and controls for percent survival with the chi-square test after transformation of percentages by the arcsine square root procedure. Deviations from a 1:1 sex ratio were analyzed by the replicated goodness-of-fit test (G-test) followed by the unplanned test of the homogeneity of replicates. In treatments were no males remained after exposure, we added 0.05 to values for all males and females for all treatments so that the natural logarithm could be calculated. Throughout all experiments, each fish was treated as a replicate for both aromatase activity and *cyp19b* expression (*n* = 6) because space for individual experimental units was limited. We compared *cyp19b* expression and aromatase activity among treatments by one-way analysis of variance (ANOVA). A Kolmogorov-Smirnov one-sample test was used to test for normality, and Levine’s test was used to test for homogeneity of variance. If data failed either test, the we used the nonparametric Kruskal-Wallis ANOVA to examine differences. If significant difference among groups was observed, a Dunn’s multiple comparisons test on each day separately was used to determine where significance occurred. Statistical significance was accepted at *p* < 0.05. All statistical analyses and graphing were completed using Sigmastat 3.1 (Systat Software, Inc., Point Richmond, CA) and SPSS 11.5 (SPSS, Inc., Chicago, IL).

## Results

### Juvenile medaka exposure.

In the first exposure, juvenile medaka were exposed in a flow-through system to FAD and TBT for 2 weeks beginning at hatch. Measured concentrations of the test chemicals were approximately 90–141% of nominal concentrations. Survival ranged from 51 to 63%, and according to the goodness-of-fit chi-square test, no significant difference in survival between controls and exposed groups existed (Table 2). Developmental exposure to FAD and TBT did not significantly alter adult sex ratios at concentrations chosen. In d-rR medaka, white males and red females indicate a phenotypic sex inversion. At this exposure, there were no sex inversions as evidenced by the lack of any white males or red females (Table 3).

In the second exposure, medaka were exposed to FAD, TBT, and *o,p*′-DDT for 2 weeks beginning at hatch. Measured doses ranged between 81 and 101% of nominal concentrations. Survival ranged from 39 to 50%, and there was no significant difference in survival between controls and exposed groups (Table 4). According to a goodness-of-fit G-test, developmental exposure to FAD and TBT did not alter sex distributions or induce sex inversion. However, *o,p*′-DDT significantly altered sex distributions in all *o,p*′-DDT exposures regardless of inhibitor cotreatment (Table 5).

### Aromatase expression.

Gene expression was measured in individual whole fry on days 5, 9, and 14 for each treatment and quantitated using the Δ Δ*C_t_* method. 18S was used as the internal normalization standard, and expression data for each time point are expressed as fold change relative to the mean of the same-day controls. Ovarian aromatase was not detected in any sample through measurement with real-time PCR or traditional PCR. Further multiple primer sets were tested in multiple conditions (data not shown). Therefore, all following data represent brain aromatase expression.

**Figure 1:**
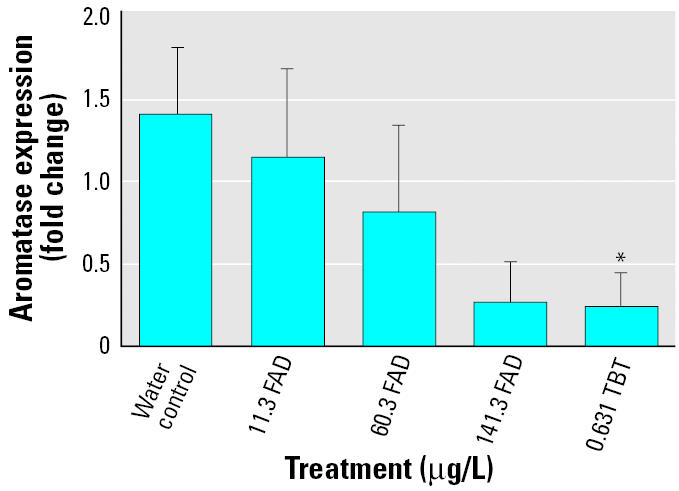
Effects of FAD and TBT on brain aromatase expression on day 5 shown as fold change relative to same-day control as measured by real-time PCR (mean ± SE). *Significant difference (*p* < 0.05).

In the first exposure, day 9 samples were lost because of defective extraction reagent. A Kruskal-Wallis ANOVA demonstrated that brain aromatase expression levels showed a significant decrease in the TBT treatment versus control treatment at sampling day 5 (Figure 1). However, this significance was lost by sampling day 14 (data not shown).

**Figure 2:**
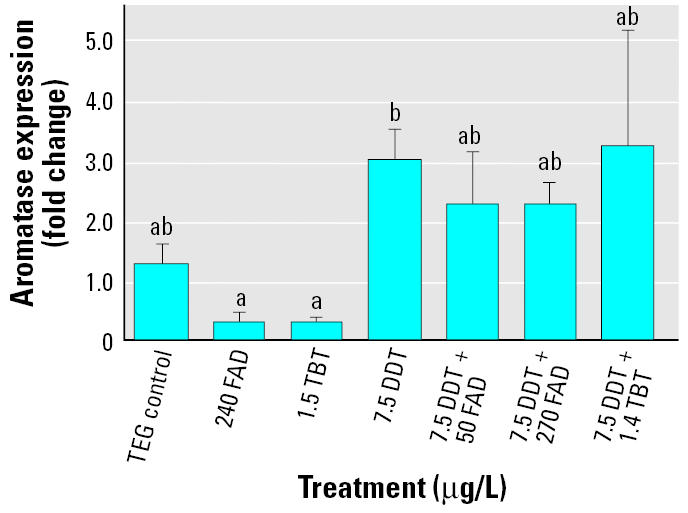
Effects of FAD, TBT, and *o,p*′-DDT, as well as FAD/DDT and TBT/DDT coexposure, on brain aromatase expression on day 14 shown as fold change (± SE) relative to same-day control as measured by real-time PCR. Bars with different letters are significantly different from each other based on Dunn’s multiple comparison test (*p* < 0.05).

In the second exposure, the day 14 sampling period demonstrated a significant difference in *cyp19b* expression between treatments according to a Kruskal-Wallis ANOVA. Pairwise multiple comparisons between treatments revealed that this difference is between FAD- and TBT-only treatments and DDT only (Dunn’s method, *p* < 0.05). Fish exposed to FAD- and TBT-only treatments also had lower expression levels than those exposed to the AI and *o,p*′-DDT cotreatments; however, these differences are not significant (Figure 2). Day 5 and day 9 sampling periods show similar trends but are not significant (results not shown).

### Aromatase activity.

**Figure 3:**
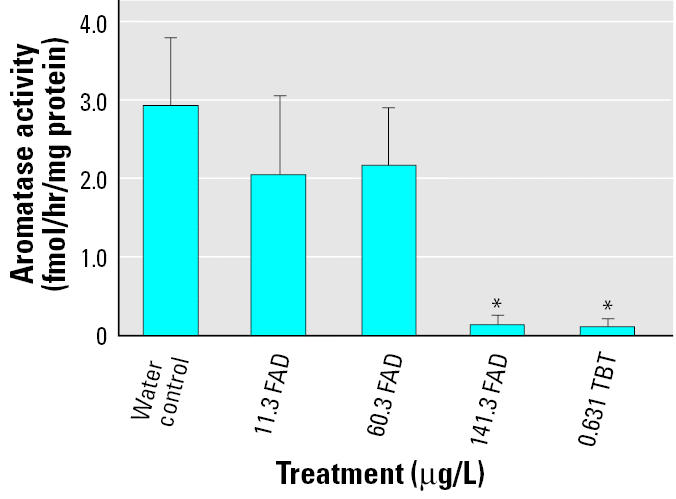
Effects of FAD and TBT exposure on aromatase enzyme activity (mean± SE, fmol/hr/mg protein) on day 14. Values for 141.3 FAD and 0.631 TBT were < LOD. *Significant difference (*p* < 0.05).

Aromatase activity was measured using a tritiated water release assay. In the first exposure, assay sensitivity was 1.49, 1.20, and 0.86 fmol/hr/mg protein for sampling days 5, 9, and 14, respectively. The highest concentration of FAD and TBT treatment levels consistently resulted in aromatase activity levels below level of sensitivity for this assay and can be considered non-detectable (Figure 3).

In the first experiment, changes in activity followed the same pattern as changes in gene expression on all sampling days, with decreasing activity at increasing concentrations of FAD and in the TBT treatment. Although day 5 and day 9 showed no significant difference between any treatments (results not shown), day 14 showed a significant decrease in activity between the controls and the highest concentrations of FAD and TBT (Figure 3).

**Figure 4:**
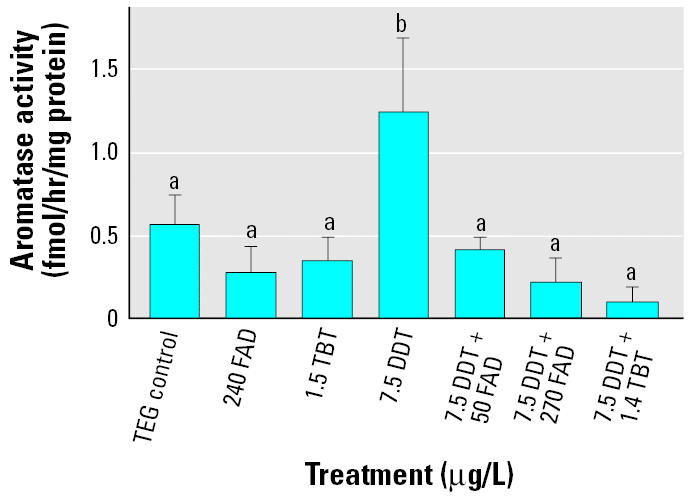
Effects of FAD, TBT, *o*,*p*′-DDT, FAD/DDT, and TBT/DDT on aromatase enzyme activity (mean ± SE, fmol/hr/mg protein) on day 14. Bars with different letters are significantly different from each other (*p* < 0.05).

For the second exposure, the limit of detection (LOD) was 2.30, 1.37, and 0.26 fmol/hr/mg protein for days 5, 9, and 14, respectively. Only the control treatment had measurable levels of aromatase activity on day 5, and all day 9 samples were < LOD. On day 14, only the *o,p*′-DDT/high FAD and *o,p*′-DDT/TBT cotreatments had enzyme activity levels below assay sensitivity (Figure 4).

Aromatase activity demonstrated a pattern different from aromatase expression. Unlike *cyp19b* expression data, which showed an increase in aromatase expression in all *o,p*′-DDT treatments regardless of presence of inhibitors, aromatase activity showed an increase only in the *o,p*′-DDT control treatment (Figure 4). A Kruskal-Wallis test demonstrated a significant difference in aromatase activity between treatments on day 14. Pairwise multiple comparison procedure revealed that the *o,p*′-DDT–only treatment is significantly different from all other treatments. The cotreatments of *o,p*′-DDT and AIs had reduced or nondetectable aromatase activity with no corresponding decrease in aromatase gene expression. However, this decrease in activity was not significant. This trend was not observed on sampling day 5 or day 9 (results not shown).

## Discussion

Most laboratory research to date on EDCs has focused on the effects of a single compound on an organism. However, in the environment, organisms are exposed to complex mixtures of potentially synergistic and antagonistic compounds, and it is unknown how these chemicals interact *in vivo* and whether such interactions diminish or exacerbate their individual effects on the health of the organism. To begin addressing these questions, our objectives in the present study were 2-fold: to examine the role of brain aromatase in *o,p*′-DDT–induced sex reversal in medaka and to determine if an environmentally relevant mixture of both estrogenic (*o,p*′-DDT) and apparent antiestrogenic (TBT) chemicals, which have opposing effects on aromatase activity, can block each other’s effects on reproductive development. Extensive experiments showed that ovarian aromatase transcripts could not be detected in medaka fry (0–14 dph) by real-time or traditional RT-PCR. Further, ovarian aromatase could not be detected upon stimulation of the estrogen response system after exposure to estradiol and *o,p*′-DDT. It appears, therefore, that the brain isoform of aromatase accounts for most, if not all, estradiol production during this life stage of the medaka and that the aromatase activity measured in this study represents the brain form of this enzyme.

To accomplish our stated objectives, we exposed juvenile medaka to an environmental estrogen in the presence and absence of two AIs and examined the effects on aromatase expression and activity and sex reversal. After a 2-week exposure, all fish developed female secondary sex characteristics regardless of the presence of AIs and independent of levels of brain aromatase activity, indicating that an increase in brain aromatase activity, as observed with *o,p*′-DDT exposure only, is not required for a male-to-female sex reversal resulting from exposure to an environmental estrogen. The results of the study presented here also show that the effects (feminization) of an environmental estrogen (*o,p*′-DDT) are not negated by the antagonistic effects of an environmental antiestrogen (TBT) at the exposure conditions and developmental stage examined. Whether the results of this specific case are of general validity for mixtures with predicted antagonistic effects deserves further study.

Additionally, results from this study demonstrate that immersion exposure to AIs alone did not result in any female-to-male inversions, even though aromatase activity was inhibited. These results contrast with several published studies in which inhibition of aromatase by both pharmaceutical (FAD) and environmental (TBT) AI exposure (McAllister and Kime 2003; Uchida et al. 2004) can result in masculinization of several fish species, including Japanese flounder, zebrafish, and salmon (Kitano et al. 2000; Kwon et al. 2000; Piferrer et al. 1993; Uchida et al. 2004). Masculinization occurs concomitantly with a decrease in aromatase expression, suggesting that manipulation of the aromatase system during development can alter gonadal differentiation (Fenske and Segner 2004).

These studies on the role of aromatase in sex reversal during development support the theory that androgens and estrogens are the natural sex inducers in fish and play pivotal roles in sex differentiation (Devlin and Nagahama 2002). Other evidence such as sexually dimorphic expression of aromatase in developing zebrafish (Trant et al. 2001) and the ability of exogenous steroid treatment to influence sex differentiation (Yamamoto 1958) suggest the importance of sex steroid production in the very early steps in gonad differentiation. Also, in medaka, inhibition of aromatase can result in the prevention of ovarian cavity formation, suggesting the importance of endogenous estrogen in gonadal development (Suzuki et al. 2004).

However, other evidence exists that suggests sex steroids may not play such an important role. For example, in medaka, germ cell differentiation appears to occur before somatic steroid-producing cells are observed (Satoh 1974). Further, in a study by Kawahara and Yamashita (2000), medaka eggs incubated with an AI resulted in no abnormal sex ratios. From these observations, the authors concluded that female sex determination in medaka is not estrogen dependent. They also suggested that estrogen-independent activation of the estrogen receptor may be a primary pathway in female gonadal development. They emphasized that the observations of female-to-male sex inversion after treatment with AI in other vertebrates and fish suggests a role of aromatase only during the sex determination period and not the importance of estrogen and aromatase as the natural sex inducer in gonadal differentiation.

In the present study, exposure to AIs significantly reduced aromatase activity between days 9 and 14 yet did not induce a sex inversion. This could be because male sex differentiation is irreversibly determined before day 9. However, it is more likely that our treatment period terminated too early, because male germ cell proliferation does not occur until 14 dph (Satoh 1974). Sensitivity of sex differentiation to endocrine disruptors has been shown to be dependent on duration and dose of exposure and on developmental stage. Insufficient exposure length or dosage concentrations can result in lack of response (Cheek et al. 2001b). The lack of response to aromatase inhibition reported by Kawahara and Yamashita (2000) may therefore be due to the developmental period of exposure. In most cases, the most sensitive period is just before or at the same time as histologic differentiation of the primitive gonad (Hunter and Donaldson 1983). In medaka, differentiation occurs at hatch, with the proliferation of ovarian cells beginning shortly after hatch (about 6–10 dph) (Satoh 1974). Medaka have been shown to be sensitive to feminization by estradiol during this proliferation period (Cheek et al. 2001a; Nimrod and Benson 1998). Unlike female germ cells, male germ cells cease to divide immediately after hatching, and proliferation is delayed until about the 9–10 mm larval stage (about 14–20 dph) (Satoh 1974). This is also the stage of development in which male gonial cells first appear in the female gonad upon androgen treatment (Kobayashi and Hishida 1985). Thus, lack of masculinization in response to aromatase inhibition observed in this study might be due to termination of exposure before male germ cell proliferation begins.

In the present study, the inhibition of aromatase did not induce a female-to-male inversion, i nor did it prevent a male-to-female inversion induced by *o,p*′-DDT. Several studies have demonstrated a correlation between an increase in aromatase expression and activity upon exposure to environmental estrogens and sex differentiation in fish (Chiang et al. 2001; Fenske and Senger 2004;Kitano et al. 2000; Scholz and Gutzeit 2000; Tanaka et al. 1995). Previous work in this laboratory (Kuhl et al. 2005) also demonstrated the importance of the aromatase system in fish gonadal development by observing a significant 5-fold increases in aromatase expression and activity at *o,p*′-DDT concentrations that induce a male-to-female sex inversion.

Results from the present study, however, demonstrate that sex inversion can be induced without a corresponding increase in aromatase activity. Treatment with *o,p*′-DDT alone induces both aromatase activity and brain aromatase expression, while resulting in a complete male-to-female sex inversion. However, inhibition of aromatase activity in *o,p*′-DDT/AI–cotreated fish also results in a complete male-to-female sex inversion. In these fish, there is an increase in brain aromatase expression while activity is reduced to nondetectable levels. Altered aromatase activity levels are therefore not a requirement for sex inversion in fish.

The apparent lack of aromatase involvement suggests that alternative, non-aromatase-dependent pathways exist through which *o,p*′-DDT may bring about sex reversal. For example, exogenous estrogen treatment can also result in reduction of expression of several steroidogenic enzymes, including P450c17, 3βHSD, and P45011β in the differentiating testis (Govoroun et al. 2001). This would decrease the synthesis of 11-oxygenated androgen and may be an important step in exogenous estrogen feminization. Further, xenobiotics can induce steroid-metabolizing enzymes, including steroid sulfotransferases, steroid glucuronidation enzymes, and steroid hydroxylases (You 2004). Because the ratio of androgens to estrogens may be more important in differentiating gonads than their absolute values (Bogart 1987), xenobiotic alterations of androgen levels may also play a role in the cotreatments conducted in this study. Steroidogenic enzyme levels and androgen to estrogen ratios during cotreatments must be measured to examine the possibility of this mechanism. Finally, non-genomic actions mediated through membrane-bound hormone receptors also can influence steroid production. Loomis and Thomas (2000) demonstrated that estrogens, and likely xenoestrogens including *o,p*′-DDT, can cause a decrease in gonadotropin-stimulated androgen production through a nongenomic mechanism in the Atlantic croaker (*Micropogonias undulatus*). This may further interfere with steroid production and the ratios between androgens and estrogens.

In conclusion, the present study demonstrates that the xenoestrogen *o,p*′-DDT increases brain aromatase activity accompanied by complete male-to-female sex inversion. Coexposure to *o,p*′-DDT and AIs does suppress aromatase activity but does not prevent sex reversal. Thus, increased aromatase activity is not necessary for sex inversion, and alternatively, aromatase-independent pathways for sex reversal resulting from xenobiotic treatment must exist. This study shows that exposure to AIs during days 0–14 fails to induce female-to-male sex inversion. This suggests that testis differentiation in d-rR medaka does not occur until after 14 dph. The observation that exposure to a mixture of estrogenic and antiestrogenic compounds does not block estrogen-induced sex reversal suggests that, in the environment, exposure to antagonistic EDCs may not necessarily lessen the harmful impacts of these compounds.

## Figures and Tables

**Table 1:** Oligonucleotide sequence for real-time PCR primers and probes.

Oligo	Sequence	Location	Function
1	AGAAGAAAACCATCCCTGGAC	208–229	*cyp19a* forward 1
2	CCATTTCCTTCCTCTTTCGCT	847–868	*cyp19a* reverse 1
3	ACTGTAGGACTCCCATCCG	120–139	*cyp19a* forward 2
4	TCTTCTCACTGTGACTCCAGA	193–214	*cyp19a* reverse 2
5	CTGCACCCTGAACAGCATCAG	94–115	*cyp19b* forward
6	AGTAGAGGGCTATCACAAAATCTC	171–192	*cyp19b* reverse
7	FAM-CCTGTCTCAGCAGCCTGTGGA-BHQ	126–152	*cyp19b* probe
8	GTTATTCCCATGACCCGCCG	1,445–1,465	18S forward
9	TTCCCGTGTTGAGTCAAATTAAGC	1,344–1,363	18S reverse
10	Cy5-ACTCCTGGTGGTGCCCTTCCGT-BHQ	1,400–1,425	18S probe

**Table 2:** Nominal and actual FAD and TBT exposure concentrations, growout survival, and water quality parameters.

Nominal (μg/L)	Actual (μg/L ± SE)	Percent nominal	Percent survival growout	Temperature (°C ± SE)	pH ± SE	DO (mg/L ± SE)
0	—		57	26.7 ± 0.1	8.8 ± 0.03	6.3 ± 0.05
10 FAD	11.29 ± 2.27	113	58	26.7 ± 0.1	8.8 ± 0.02	6.5 ± 0.05
50 FAD	60.25 ± 0.5	121	63	26.7 ± 0.1	8.7 ± 0.04	6.2 ± 0.05
100 FAD	141.3 ± 5.81	141	52	26.8 ± 0.1	8.7 ± 0.02	6.3 ± 0.04
0.7 TBT	0.63 ± 0.007	90	51	26.7 ± 0.1	8.7 ± 0.04	6.3 ± 0.05

DO, dissolved oxygen.

**Table 3:** Sex and color at sexual maturity after FAD and TBT treatments.

Treatment	Red genotypic male	White phenotypic male	White genotypic female	Red phenotypic female
Control	32	0	35	0
11.3 FAD	35	0	34	0
60.3 FAD	36	0	38	0
141 FAD	27	0	34	0
0.63 TBT	28	0	32	0

**Table 4:** . Nominal and actual FAD, TBT, and DDT exposure concentrations with growout survival.

Treatment	Nominal (μg/L)	Actual TBT (μg/L ± SE)	Actual FAD (μg/L ± SE)	Actual *o*,*p*′-DDT (μg/L ± SE)	Percent survival growout
TEG control	0	—	—	—	40
FAD	300	—	243 ± 6.7	—	40
TBT	1.5	1.52 ± 0.03	—	—	44
DDT	7.5	—	—	7.48 ± 0.04	38
DDT + low FAD	7.5 + 50	—	49.75 ± 11.6	7.42 ± 0.05	40
DDT + high FAD	7.5 + 300	—	270 ± 7.6	7.49 ± 0.04	50
DDT + TBT	7.5 + 1.5	1.40 ± 0.03	—	7.53 ± 0.08	39

Abbreviations: —, not applicable; TEG, triethylene glycol.

**Table 5:** Sex and color at sexual maturity in *o*,*p*′-DDT/FAD and *o*,*p*′-DDT/TBT cotreatments.

Treatment	Red genotypic male	White phenotypic male	White genotypic female	Red phenotypic female
TEG control	24	0	23	0
FAD	18	0	28	0
TBT	26	0	24	0
DDT	0	0	21	22
DDT + low FAD	0	0	26	20
DDT + high FAD	0	0	33	25
DDT + TBT	0	0	25	19

TEG, triethylene glycol.
